# miR-135b-5p inhibits LPS-induced TNFα production via silencing AMPK phosphatase Ppm1e

**DOI:** 10.18632/oncotarget.12866

**Published:** 2016-10-25

**Authors:** Ping Li, Jian-bo Fan, Yanxia Gao, Ming Zhang, Li Zhang, Ning Yang, Xiaojing Zhao

**Affiliations:** ^1^ Department of Emergency, the Second Affiliated Hospital of Xi'an Jiao Tong University, Xi'an, China; ^2^ Department of Orthopaedics, The Second Affiliated Hospital of Nantong University, Nantong, China

**Keywords:** miR-135b-5p, Ppm1e, AMPK, LPS, TNFα

## Abstract

AMPK activation in monocytes could suppress lipopolysaccharide (LPS)-induced tissue-damaging TNFa production. We are set to provoke AMPK activation via microRNA (“miRNA”) downregulating its phosphatase Ppm1e. In human U937 and THP-1 monocytes, forced expression of microRNA-135b-5p (“miR-135b-5p”) downregulated Ppm1e and activated AMPK signaling. Further, LPS-induced TNFα production in above cells was dramatically attenuated. Ppm1e shRNA knockdown in U937 cells also activated AMPK and inhibited TNFα production by LPS. AMPK activation is required for miR-135b-induced actions in monocytes, AMPKα shRNA knockdown or T172A dominant negative mutation almost abolished miR-135b-5p's suppression on LPS-induced TNFα production. Significantly, miR-135b-5p inhibited LPS-induced reactive oxygen species (ROS) production, NFκB activation and TNFα mRNA expression in human macrophages. AMPKα knockdown or mutation again abolished above actions by miR-135b-5p. We conclude that miR-135b-5p expression downregulates Ppm1e to activate AMPK signaling, which inhibits LPS-induced TNFα production via suppressing ROS production and NFκB activation.

## INTRODUCTION

Chronic obstructive pulmonary disease (COPD) patients suffer chronic and consistent airway inflammations [1–3]. A significant increased level of circulating pathogen-associated molecular patterns (PAMPs) is detected in COPD patients’ lungs [1–3]. Lipopolysaccharide (LPS), one of the most prominent PAMPs, activates resident monocytes and induces production pro-inflammatory cytokines (*i.e.* tumor necrosis factor-α or TNFα) [4, 5]. TNFα level is significantly elevated in COPD patients’ bronchoalveolar lavage fluids, sputum, as well as plasma and lung tissues [6–8]. Anti-TNFα strategy was applied to attenuate COPD patients’ inflammations [6–8]. Our group has been focusing on the underlying mechanisms of LPS-induced TNFα production in monocytes [9], which might help to develop possible intervention measures [9].

AMP-activate protein kinase (AMPK) plays a pivotal role in maintaining cellular energy balance [10]. Recent studies have discovered the important function of this kinase in regulating inflammatory responses [11–14]. For instance, two well-known AMPK activators, AICAR and A769662, were shown to inhibit LPS-induced nuclear factor kappa B (NFκB) activation and pro-inflammatory cytokine production [11, 12]. Ducommun *et al.*, showed that metformin activated AMPK signaling to inhibit cytokine-induced pro-inflammatory responses [15]. Shen *et al.*, showed that perifosine surprisingly reduced LPS-induced TNFα production via activating AMPK [12]. Our unpublished results recently found that GSK621, a novel AMPK activator [16], inhibited LPS-induced TNFα production in macrophages/monocytes (Wu *et al.*, unpublished results). Therefore, targeted-activation of AMPK could be a novel strategy to inhibit LPS-induced inflammatory responses [11, 12, 15, 17].

Thr172 phosphorylation of AMPK α subunit is vital for AMPK activation [10, 18, 19]. Numerous studies have focused on the mechanisms of kinase phosphorylation of this site [20]. Several AMPK kinases (*i.e.* LKB1 [19], CaMKK [21] and TAK1 [22]) have been characterized thus far. Yet, the phosphatase of AMPKα-Thr172 is largely unknown. A recent study by Voss *et al.*, has proposed that Ppm1e could be a key AMPKα phosphatase [23]. Ppm1e depletion, inhibition or mutation was able to induce AMPKα-Thr172 phosphorylation and AMPK activation [23].

microRNAs (miRNAs) are capable of decreasing expression of target mRNAs at both post-transcriptional and transcriptional levels [24]. Here we set to indentify miRNA that activates AMPK though specifically targeting Ppm1e. Multiple miRNA databases were searched. We found that microRNA-135b-5p (“miR-135b-5p”) selectively targets Ppm1e's untranslated regions (UTRs, 3′). Further, forced-expression of miR-135b-5p downregulates Ppm1e to activate AMPK signaling, which subsequently inhibits LPS-induced TNFα production in human monocytes.

## RESULTS

### Expression of miR-135b-5p downregulates Ppm1e in human macrophages

**Figure 1:**
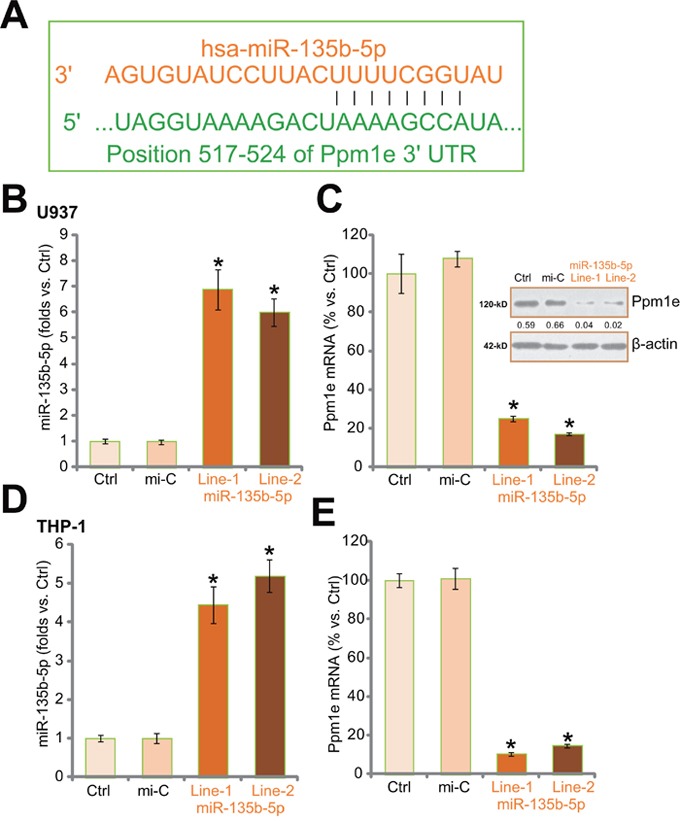
Expression of miR-135b-5p downregulates Ppm1e in human macrophages **A.** miR-135b-5p complements Ppm1e's 3′ untranslated regions (UTRs, position 517-524) (A). Human U937 or THP-1 macrophages were transfected with miR-135b-5p construct or non-sense control microRNA (“miR-C”), and stable cells were established via neomycin selection. Expression of miR-135b-5p **B** and **D**. and Ppm1e mRNA (**C** and **E.**, left panels) were tested by quantitative real-time PCR (“RT-qPCR”) assay; Ppm1e protein expression was examined by Western blot assay (C, right panels). Experiments in this figure were repeated three times, and similar results were obtained. Ppm1e protein expression (vs. β-actin) was quantified (C). “Ctrl” stands for non-transfected control cells (B-E). *p<0.05 vs. “miR-C” group (B-E).

First, miR-135b-5p indeed complements UTRs (position 517-524) of Ppm1e (Figure 1A, which is also seen in the recent study [25]). As described, an expression vector (“pSuper-neo”) integrating miR-135b-5p [25] was transfected to U937 macrophages. Via neomycin selection, two stably U937 lines (“Line-1” and “Line-2”) with miR-135b-5p were established. Real-time qPCR (“RT-qPCR”) assay analyzing miR-135b-5p level confirmed the phenotype of two stably cell lines, with high level of miR-135b-5p expression (Figure 1B). Intriguingly, mRNA and protein expressions of Ppm1e were sharply downregulated following miR-135b-5p expression (Figure 1C). The experiments were also repeated in another human macrophage cell line: THP-1. Two stably THP-1 cell lines (“Line-1” and “Line-2”) expressing miR-135b-5p (Figure 1D) were established. These cell lines again showed downregulated Ppm1e (Figure 1E). Notably, the non-sense control mi-RNA (“miR-C”) showed no effect on expression of miR-135b-5p (Figure 1B and 1D) or Ppm1e (Figure 1C and 1E). Together, these results demonstrate that miR-135b-5p targets and downregulates Ppm1e in human macrophages.

### miR-135b-5p activates AMPK and inhibits LPS-induced TNFα production in human macrophages

**Figure 2:**
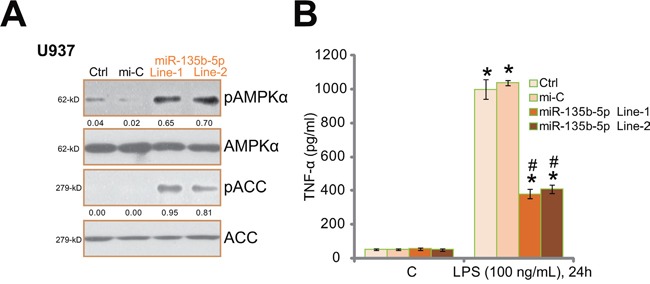
miR-135b-5p activates AMPK signaling and inhibits LPS-induced TNFα production in human macrophages Stably U937 macrophages expressing miR-135b-5p (two lines, “Line-1/-2”) or non-sense control microRNA (“miR-C”) were subjected to Western blot assay of phosphorylated- (“p”) and regular AMPKα and ACC **A.** Above cells were treated with LPS (100 ng/mL) or medium control (“C”) for 24 hours, TNFα content in conditional medium was tested by ELISA assay **B.** Experiments in this figure were repeated for three times, and similar results were obtained. AMPKα and ACC phosphorylations were quantified (A). “Ctrl” stands for un-transfected control cells. * *p*<0.05 vs. group “C” (B). ^#^ *p*< 0.05 *vs.* LPS treatment of “miR-C” group (B).

Previous studies have demonstrated that Ppm1e is an AMPK phosphatase [23]. Above results showed that miR-135b-5p downregulated this phosphatase in human macrophages. Thus AMPK signaling was then tested in these cells. As shown in Figure 2A, the level of phosphorylated (“p”) AMPKα (Thr-172) and its major downstream acetyl-CoA carboxylase (ACC, Ser-79) was significantly increased in miR-135b-5p-expressing U937 cells, indicating profound AMPK activation. As discussed, studies have implied that AMPK activation can inhibit LPS-induced inflammatory responses [11–14]. Therefore, the potential effect of miR-135b-5p on LPS-induced TNFα protein was then tested. ELISA assay results in Figure 2B showed that LPS-induced TNFα protein secretion was largely inhibited in miR-135b-5p-expressing U937 cells. miR-135b-5p resulted in over 60% reduction of TNFα production (Figure 2B). Similar results were also obtained in THP-1 macrophages, where miR-135b-5p induced significant AMPK activation and inhibited LPS-induced TNFα production (Data not shown).

### Ppm1e shRNA knockdown activates AMPK and inhibits LPS-induced TNFα production

**Figure 3:**
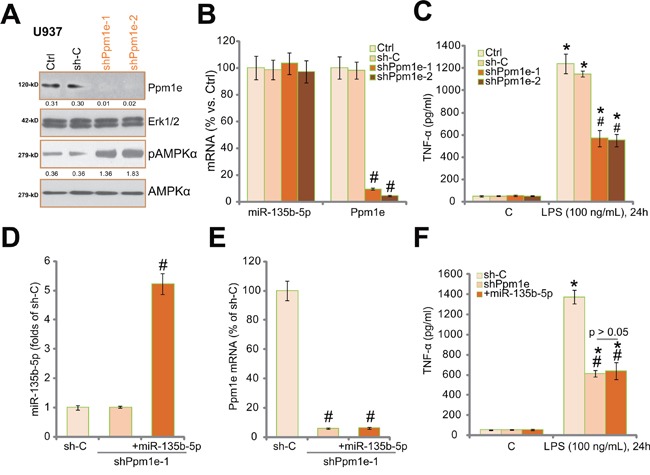
Ppm1e shRNA knockdown activates AMPK and inhibits LPS-induced TNFα production in human macrophages Stably U937 cells expressing Ppm1e shRNA (“shPpm1e-1” or “shPpm1e-2”, with non-overlapping sequences) or scramble control shRNA (“sh-C”) were subjected to Western blot assay of listed proteins **A.** or RT-qPCR assay of miR-135b-5p and Ppm1e mRNA **B.** Above cells were treated with LPS (100 ng/mL) or medium control (“C”) for 24 hours, TNFα production was tested by ELISA assay **C.** U937 cells with shPpm1e-1 were also transfected with miR-135b-5p construct, and stably cells were again established; miR-135b-5p **D.** and Ppm1e mRNA **E.** expressions were tested by RT-qPCR assay. Above cells were treated with LPS (100 ng/mL) for 24 hours, TNFα production was measured **F.** Ppm1e expression (vs. Erk1/2) and AMPKα phosphorylation were quantified (A). “Ctrl” stands for un-transfected control cells. Experiments in this figure were repeated for three times, and similar results were obtained. ^#^ *p* < 0.05 *vs.* “sh-C” group (B-F). * *p* < 0.05 *vs.* “C” group (C and F).

Based on the results above, Ppm1e knockdown should also activate AMPK and inhibit TNFα production. Thus, lentiviral shRNA strategy was applied to knockdown Ppm1e in U937 cells. Two stably U937 cell lines with Ppm1e-shRNA (“-1/-2”) were established. Western blot results in Figure 3A confirmed that Ppm1e expression was downregulated in the stably cells. Consequently, AMPK activation (p-AMPKα) was increased (Figure 3A). Notably, Ppm1e shRNA didn't change miR-135b-5p expression (Figure 3B). Significantly, LPS-induced TNFα production in U937 cells was dramatically attenuated with Ppm1e shRNA knockdown (Figure 3C). The scramble non-sense control shRNA (“sh-C”) showed no effect on Ppm1e expression, AMPK activation nor TNFα production (Figure 3A and 3B). We repeated the above experiments in THP-1 cells, and similar results were achieved (Data not shown).

If, as we proposed, Ppm1e is the primary target of miR-135b-5p in mediating its actions in monocytes, miR-135b-5p should possibly be invalid in Ppm1e-depleted cells. We thus expressed miR-135b-5p in Ppm1e-shRNA expressing U937 cells. RT-qPCR assay results confirmed miR-135b-5p over-expression (Figure 3D) in the Ppm1e-silence U937 cells (Figure 3E). Importantly, miR-135b-5p expression failed to further inhibit LPS-induced TNFα production in the Ppm1e-silenced cells (*p* > 0.05, Figure 3F). These results indicate that Ppm1e is likely the primary target of miR-135b-5p in mediating its actions against LPS.

### AMPK activation is required for miR-135b-5p's inhibition on LPS-induced TNFα production

**Figure 4:**
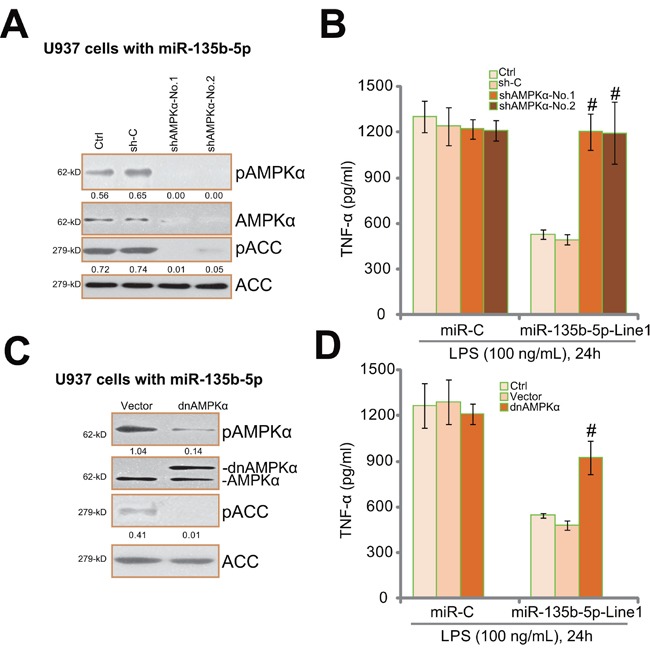
AMPK activation is required for miR-135b-5p's inhibition on LPS-induced TNFα production miR-135b-5p expressing U937 cells were constructed with AMPKα shRNA (“shAMPKα-No.1”/“shAMPKα-No.2”) or scramble control shRNA (“sh-C”), and stably cells were established; Expressions of listed proteins in these cells were tested by Western blot assay **A.**; Cells were treated with LPS (100 ng/mL) for 24 hours, TNFα production was tested **B.** miR-135b-5p expressing U937 cells were constructed with dominant negative AMPKα (T172A, “dnAMPKα”, GFP-tagged) or empty vector (“pSuper-puro”), stably cells were established; Expressions of listed proteins in these cells were tested by Western blot assay **C.**; LPS-induced TNFα production was also examined **D.** Experiments in this figure were repeated for three times, and similar results were obtained. AMPKα and ACC phosphorylations were quantified (vs. regular ACC, A and C). “Ctrl” stands for un-transfected control cells (B and D). ^#^ *p* < 0.05 *vs.* “sh-C” (B). ^#^ *p* < 0.05 *vs.* “Vector” (D).

If AMPK activation is the primary reason of miR-135b-5p-induced action against TNFα production by LPS, AMPK inhibition should then abolish miR-135b's activity. Genetic strategies were applied. Two different non-overlapping lentiviral AMPKα shRNAs (“No.1” and “No.2”) were utilized to knockdown AMPKα in miR-135b-5p-expressing U937 cells (Figure 4A). As a result, miR-135b-5p-induced AMPK activation, or AMPKα/ACC phosphorylation, was dramatically inhibited (Figure 4A). Remarkably, AMPKα shRNAs almost abolished miR-135b-5p-induced inhibition of TNFα production (Figure 4B). In another words, miR-135b-5p was in-effective against TNFα production in AMPKα-depleted cells (Figure 4B). Above results suggest that AMPK activation is required for miR-135b-5p-induced actions in monocytes. Net, we introduced a dominant-negative mutant of AMPKα (T172A) into miR-135b-5p-expressing U937 cells [26]. The mutant AMPKα dramatically attenuated AMPK activation (Figure 4C). Significantly, ELISA results in Figure 4D showed that AMPKα mutation almost nullified miR-135b's inhibition on TNFα production by LPS. These two sets of results clearly indicate that miR-135b-5p inhibits LPS-induced TNFα production through activating AMPK.

### miR-135b-5p inhibits LPS-induced ROS production, NFκB activation and TNFα mRNA expression

**Figure 5:**
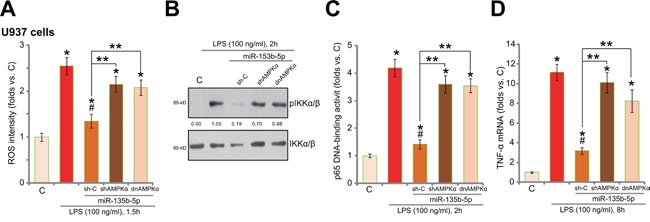
miR-135b-5p inhibits LPS-induced ROS production, NFκB activation and TNFα mRNA expression miR-135b-5p expressing U937 cells were constructed with scramble control shRNA (“sh-C”), AMPKα shRNA (“shAMPKα”, No.1) or dominant negative AMPKα (T172A, “dnAMPKα”), these cells or the control U937 cells were treated with LPS (100 ng/mL) or medium control (“C”) for applied time, relative ROS intensity **A.,** NFκB activation **B** and **C.** and TNFα mRNA expression **D.** were tested by listed assays. Experiments in this figure were repeated for three times, and similar results were obtained. IKKα/β phosphorylation was quantified (B). *p < 0.05 v*s.* “C” group (A, C and D). ^#^ *p* < 0.05 *vs.* LPS only group (A, C and D). ** *p* < 0.05 (A, C and D).

Existing evidences have shown that LPS induces production of reactive oxygen species (ROS), which is required for subsequent NFκB activation and TNFα mRNA expression [17, 27]. Interestingly, AMPK activation may function as an anti-oxidant signaling within a number of stress conditions [17, 26, 28–30]. We therefore analyzed ROS level and NFκB signaling in human macrophages with/our miR-135b-5p expression. As shown in Figure 5A, treatment of LPS in U937 cells induced significant ROS production, which was largely attenuated with miR-135b-5p expression (Figure 5A). Intriguingly, miR-135b-5p-induced anti-oxidant function also relies on AMPK activation (Figure 5A). AMPKα shRNA knockdown or mutation almost abolished miR-135b-5p's ROS scavenging activity (Figure 5A).

Intriguingly, LPS-induced NFκB activation, tested by Western blot assay of pIKKα/β (Ser176/180) (Figure 5B) and p65 DNA-binding assay (Figure 5C) [9], was significantly inhibited with miR-135b-5p expression. Consequently, LPS-induced TNFα mRNA expression was also attenuated (Figure 5D). Such effects by miR-135b-5p were again almost abolished with AMPKα knockdown or mutation (Figure 5B-5D). Collectively, these results indicate that miR-135b-5p activates AMPK to inhibit LPS-induced ROS production, NFκB activation and TNFα mRNA expression.

## DISCUSSION

LPS is sensed by CD14 and LPS-binding protein (LBP), and binds to its receptor Toll-like receptor 4 (TLR-4) on monocytes [31, 32]. This will lead to the recruitment of several key adaptor proteins (MyD88, TRAF6 and others) to activate downstream NFκB signaling cascade [31, 32]. ROS production also plays a pivotal role in the process. Sanlioglu *et al.*, showed Rac1-dependent ROS production induced LPS-induced NFκB activation and TNFα production [27]. On the other hand, ROS scavengers could attenuate LPS-induced inflammatory response [27]. For instance, Shen *et al.*, demonstrated that cordycepin inhibited LPS-induced ROS production and subsequent TNFα production [17]. In the present study, we showed that miR-135b-5p activated AMPK signaling to inhibit LPS-induced ROS production and subsequent NFκB activation. This could be one key reason of TNFα inhibition by miR-135b-5p.

Existing evidences have implied AMPK as an anti-oxidative signaling under a number of stress conditions [17, 29, 30]. AMPK activation by energy depletion could attenuate oxidative stress via increasing NADPH content [30]. In this regard, AMPK-ACC signaling activation inhibits ROS accumulation via increasing NADPH production [30]. A recent study by She *et al.*, demonstrated that AMPK activation could decrease H_2_O_2_-induced oxidative damages [29]. Recently, Zhang's group showed that cordycepin suppressed LPS-induced ROS production and NFκB activation through activating AMPK-NADPH signaling [17]. Our recent unpublished work showed that GSK621, a novel AMPK activator [16], attenuated LPS-induced ROS production, NFκB activation and subsequent TNFα expression (Wu et al., unpublished studies). In line with these findings, we show that AMPK activation by miR-135b-5p decreased LPS-induced ROS production and NFκB activation. Such effects by miR-135b-5p were almost abolished with AMPK inhibition. Thus, we propose that miR-135b-5p activates AMPK to attenuate LPS-induced ROS production, and subsequent NFκB activation, which then inhibit TNFα mRNA expression and production.

Although many AMPK activators have been developed thus far [33], there are few of them are being tested in clinical stages for various disease. The results of this study showing AMPK activation by miR-135b-5p via downregulating Ppm1e provide a new strategy to activate AMPK and to inhibit LPS inflammatory responses.

## MATERIALS AND METHODS

### Chemicals and antibodies

LPS, puromycin and neomycin were purchased from Sigma Chemicals (Shanghai, China). All the antibodies utilized in this study were purchased from Cell Signaling Technology (Danvers, MA). The cell culture reagents were purchased from Hyclone of Thermo Fisher Scientific (Shanghai, China).

### Cell culture

The human monocyte cell lines, U937 and THP-1, were purchased from the Cell Bank of Fudan University (Shanghai, China). Cells were cultured in RPMI 1640 supplemented with 10% FBS and 1% *L*-glutamine at 37°C.

### Real-Time PCR assay

The protocol of real-time reverse transcriptase quantitative polymerase chain reaction (RT-qPCR) was described in detail in our previous study [9]. The comparative Ct method (2^−ΔΔCt^) was applied to calculate relative mRNA expression level [34]. Glyceraldehyde-3-phosphate dehydrogenase (GAPDH) was tested as the reference gene. The primers for TNFα (F: 5′-GGAGGGGTCTTCCAGCTGGAGA-3′, and R: 5′-CAATGATCCCAAAGTAGACCTGC-3′) and GAPDH (F: 5′-AGGCTAGCTGGCCCGATTTC-3′, and R: 5′-TGGCAACAATATCCACTTTACCAGA-3′) were utilized. The primers for Ppm1e were described in other studies [35]. The expression of mature hsa-miR-135b-5p was evaluated by the TaqMan microRNA assay as described [36]. Five ng of total RNA was reverse-transcribed using TaqMan MicroRNA Reverse Transcription Kit (Applied Biosystem, Shanghai, China) [36]. All the primers and sequences were synthesized by Genepharm (Shanghai, China).

### Forced miR-135b-5p expression

The pSuper-neo expression vector withmiR-135b-5p (based on sequence [36]) was provided by Dr. Cui's group at Nantong University (Nantong China) [25]. U937 or THP-1 cells were transfected with miR-135b-5p construct (0.10 μg/mL of each well) through the Lipofectamine 2000 protocol (Invitrogen, Shanghai, China) for 24 hours. Stably cells were then selected by neomycin (0.25 μg/mL) for two weeks. miR-135b-5p expression in the stable cells was tested by the RT-qPCR assay. Control cells were transfected with non-sense scramble microRNA-control (“miR-C”) (Also provided by Dr. Cui [25]).

### TNFα enzyme-linked immunosorbent assay (ELISA) assay

Following treatment of cells, TNFα content in the conditional medium was evaluated via the TNFα ELISA kit (R&D Systems, Abingdon, UK) as described [9].

### Western blots

As described [9], the protein lysates (20 μg per sample) were separated by SDS-PAGE gel, and were transferred onto PVDF membranes, which were then probed with primary and secondary antibodies. Enhanced chemiluminescence (ECL, Amersham, Shanghai, China) regents were utilized to detect targeted bands. The total gray of each protein band was quantified by Bio-Rad Quantity One software, and was normalized to corresponding loadings [9].

### shRNA knockdown

The two lentiviral AMPKα short hairpin RNAs (shRNAs, “No1” and “No2”, with non-overlapping sequences) were provided by Dr. Lu's group at Nanjing Medical University [37–39]. The two different Ppm1e lentiviral shRNAs (“-1/-2”) were provided by Dr. Cui's group at Nantong University. The lentiviral shRNA was added to cultured cells for 24 hours, and stably cells were selected by puromycin (1.0 μg/mL) for 10-14 days [37–39]. Knockdown of AMPKα or Ppm1e was verified by Western blot assay. Control cells were infected with non-sense control shRNA lentiviral particles (Santa Cruz Biotech).

### AMPKα dominant negative mutation

The pSuper-puro construct with dominant negative (T172A) AMPKα and the empty vector were provided by Dr. Lu's group at Nanjing Medical University [37, 40]. Lipofectamine 2000 was applied to transfect mutant AMPKα or the vector to miR-135b-expressing U937 cells. Stable cells were again selected by puromycin (1.0 μg/mL). AMPKα mutation was verified by Western blot assay.

### Reactive Oxygen Species (ROS) assay

ROS production was measured by dichlorofluorescin (DCF) oxidation assay as described [17]. Briefly, after applied treatment, cells were incubated with 10 μM of DCFH-DA (Invitrogen) for 30 min. Cells were then washed, trypsinized and resuspended in PBS. DCF fluorescence intensity was then tested using a FACS BD machine. The fluorescent intensity value of treatment group was expressed as fold changes of the control group.

### Measuring NFκB (p65) DNA-binding activity

The detailed protocol of this assay was described in our previous study [9]. Briefly, after treatment of cells, NFκB (p65) DNA-binding activity, analyzing from 1.0 μg of cell nuclear extracts, was examined using the TransAM™ ELISA kit (Active Motif, Carlsbad, CA) with the manufacturer's protocol. The OD value of treatment group was always normalized to that of control group.

### Statistics analysis

The statistical analyses were performed via the SPSS software (18.0), with *p* < 0.05 taken as significant. Data were expressed as mean ± standard deviation (SD). For comparisons among multiple groups, two-way ANOVA with the Bonferroni post hoc testing was performed.
